# 2264 Early findings from a real-world RCT: Acceptance and commitment therapy (ACT) for persistent pain in an integrated primary care setting

**DOI:** 10.1017/cts.2018.282

**Published:** 2018-11-21

**Authors:** Kathryn E. Kanzler, Patricia Robinson, Mariana Munante, Donald McGeary, Jennifer Potter, Eliot Lopez, Jim Mintz, Lisa Kilpela, Willie Hale, Donald Dougherty, Dawn Velligan

**Affiliations:** 1 University of Texas San Antonio; 2 UT Health San Antonio

## Abstract

OBJECTIVES/SPECIFIC AIMS: This study seeks to test the feasibility and effectiveness of a brief Acceptance and commitment therapy (ACT) treatment for patients with persistent pain in a patient-centered medical home. METHODS/STUDY POPULATION: Participants are recruited via secure messaging, clinic advertisements and clinician referral. Primary care patients age 18 and older with at least 1 pain condition for 12 weeks or more in duration are stratified based on pain severity ratings and randomized into (a) ACT intervention or (b) control group [Enhanced Treatment as Usual (E-TAU)]. Participants in the ACT arm attend 1 individual visit with an integrated behavioral health provider, followed by 3 weekly ACT classes and a booster class 2 months later. E-TAU participants will receive usual care plus patient education handouts informed by cognitive behavioral science. Currently, 17% of our overall goal of 60 patients have completed ACT or enhanced treatment as usual. Average participant age is 49 years old, 70% female, and 70% Hispanic/Latino. Most report multisite pain conditions (e.g., musculoskeletal, fibromyalgia) and 30% are taking opioid medications. Data analysis in this presentation will include early correlational findings from baseline assessments. Upon study completion, we will analyze data using a general linear mixed regression model with repeated measures. RESULTS/ANTICIPATED RESULTS: The overall hypothesis is that brief ACT treatment reduces physical disability in patients with persistent pain when delivered by an integrated behavioral health provider in primary care. By examining a subset of patients on opioid medications, we also anticipate a reduction in opioid misuse behaviors. Additionally, it is anticipated that improvements in patient functioning will be mediated by patient change in pain acceptance and patient engagement in values-consistent behaviors. DISCUSSION/SIGNIFICANCE OF IMPACT: This pilot study will establish preliminary data about the feasibility and effectiveness of addressing persistent pain in a generalizable, “real-world” integrated primary care setting. Data will help support a larger trial in the future. If effective, findings could improve treatment methods and quality of life for patients with persistent pain using a scalable approach.

